# Evolutionary medicine

**DOI:** 10.7554/eLife.69398

**Published:** 2021-07-22

**Authors:** George H Perry

**Affiliations:** Departments of Anthropology and Biology, and the Huck Institutes of the Life Sciences, Pennsylvania State UniversityUniversity ParkUnited States

**Keywords:** human evolutionary history, genomics, risk of disease, cancer, drug resistance, pleiotropy

## Abstract

In recognition that evolutionary theory is critical for understanding modern human health, eLife is publishing a special issue on evolutionary medicine to showcase recent research in this growing and increasingly interdisciplinary field.

Our individual and collective health is shaped and affected by many factors. These factors include our environment, our inherited and somatic genetic variants, our variable exposure to pathogens, our diets and lifestyles, our social systems, and our cultural innovations.

None of these factors are static, and they all interact with each other. Human genetic adaptations to our past environments, disease burdens, and cultural practices can affect disease risks today, especially if any of the underlying environmental, disease, or cultural factors have changed in the interim. Meanwhile, human pathogens and parasites continually adapt to our biology and to cultural innovations, including advances in medicine, the development of new drugs, and infrastructure improvements (such water-treatment plants or the availability of mosquito nets). The progression of cancer within an individual is also often viewed as an evolutionary process (Merlo et al., 2006).

Growing numbers of scientists are applying evolutionary theory to study these interactions across different timescales and their impacts on modern human health, including with predictions of how our health might be affected by these processes in the future and how we can take informed action. This field of study is known as evolutionary medicine (Stearns and Medzhitov, 2015). To help set the stage for a special issue of eLife on evolutionary medicine, I will highlight a subset of concepts and research approaches in this field.

One area of particularly active research is studying how various pathogens and parasites evolve within and among human hosts, between human and non-human hosts and/or vectors, and in response to drug treatments. Work on the evolution of bacterial pathogen resistance to antibiotics (Bakkeren et al., 2020), virus resistance to antivirals (Irwin et al., 2016), fungal resistance to antifungals (Robbins et al., 2017), and parasite resistance to other antimicrobials (e.g., Haldar et al., 2018) is understandably prominent. Yet the evolutionary forces of mutation, genetic drift and gene flow (including that mediated by human host, animal host, and insect vectors movement and behavior), and the interplay between these forces and human immunity, are also critical components of human infectious disease dynamics.

Basic and applied questions in this area of evolutionary medicine research include: How does resistance develop and spread? How repeatable is it (Igler et al., 2021)? What cultural factors – beyond the drug treatment itself – play major roles in this process? How does resistance evolve in multiple drug treatment scenarios (McLeod and Gandon, 2021), and what are the interaction effects with co-occurring pathogens? What are the best practices for treating patients, given this knowledge (Morley et al., 2020)? What features of newly designed drugs are optimal in the face of these evolutionary processes? How do other evolutionary forces acting on our pathogens impact human health, separately and in combination with resistance evolution (D’Aeth, 2021; Huddleston et al., 2020)?

Taking a longer-term view, many people today live in environments that are markedly different from those of their ancestors. This may reflect histories of migration or forced transport, climate change, and/or cultural change (such as shifts from hunting and gathering to subsistence agriculture, and to industrialization). Evolutionary ‘mismatch’ is the phenomenon whereby genetic variants that were adaptive in the past are now associated with an increased risk of disease in the context of one’s current environment (Manus, 2018). Evolutionary mismatch as an appropriately cautious concept has validity and this idea has motivated promising gene by environment interaction research (Benton et al., 2021).

However, it is important to keep in mind that evolutionary processes are continuous. That is, our individual genomes reflect complex histories of past adaptation and genetic drift across many time periods. This means that connections between past evolutionary ecology, subsequent environmental change, and modern health are not always straightforward. Take the example of diet: some argue that for optimal health modern humans should follow a ‘paleo diet’ (which is focused on consuming meat, fish, fruit and vegetables, and on avoiding grains, dairy products and processed foods). However, there is extensive scientific evidence of ongoing human biological adaptations in response to post-‘paleo’ diet-related cultural changes (Chang and Nowell, 2016; Zuk, 2013), contradicting the absolutism of the paleo diet industry.

Meanwhile, genetic variants that may have been subjected to positive selection in the past because they reduced the risk or severity of one disease may simultaneously confer increased risk for other diseases (Williams, 1957; Byars et al., 2017). As an example of such a 'pleiotropic trade-off', variants in a human gene called *APOL1* confer resistance to infection by *Trypanosoma brucei* parasites that cause African sleeping sickness. These *APOL1* variants are associated with strong signatures of past positive selection in some African populations. However, the same alleles are also associated with an increased risk for kidney disease in African Americans (Cooper et al., 2017; Genovese et al., 2010).

When a genetic signature of positive selection for an allele (or set of alleles) associated with an increased risk of a disease is identified (see, for example, Clemente et al., 2014 and Richard et al., 2020), scientists can assess whether evolutionary mismatch, pleiotropic trade-off, or both are involved. By then considering the shifting environmental context (Byars et al., 2017; Zhang and Gems, 2021) or the molecular mechanisms and epidemiology of linked diseases, our knowledge of disease biology, potential treatment pathways, and intervention approaches can grow in compounded fashion.

Evolutionary medicine research with diverse human groups can be broadly informative as each local population has its own eco-evolutionary history and potentially distinct biological adaptations. For example, an allele that was likely driven by positive selection to relatively high frequencies in Polynesians – but is rare or absent in other populations – is associated with a substantially increased risk of obesity but a decreased risk of type II diabetes, which is in contrast to what we would expect based on typical risk factor relationships (Krishnan et al., 2018; Minster et al., 2016). A more complete understanding of the underlying biological pathway in this case could lead to new strategies for treating type II diabetes.

In principle, worldwide participation in scientific research and expansive sharing in its benefits can improve equity. However, researchers must pay attention to important ethical considerations, especially when partnering with Indigenous populations (Hernandez and Perry, 2021). In particular, efforts should be made to ensure that communities are fairly compensated and receive other longer-term benefits related to the knowledge and any new treatments that are developed on the basis of their participation (Fox, 2020).

Another strand of research within evolutionary medicine is the use of comparative phylogenomic and experimental approaches to uncover the mechanisms responsible for naturally occurring variation among diverse animal taxa to help advance our understanding of human health. For example, we might expect cancer risk to be positively correlated with body size and lifespan: this is true within species, but not between species. One article in the special issue examines the duplication of tumor suppressor genes in elephants (which have relatively low cancer risk) and related, smaller-bodied mammals (Vazquez and Lynch, 2021). Other researchers have explored the molecular underpinnings of unexpected (relative to body size) longevity in bats (Seim et al., 2013), and oxidative stress resistance and longevity in naked mole rats (Fang et al., 2014; Kim et al., 2011; Takasugi et al., 2020).

Given that research in evolutionary medicine can involve the intersection of human health, cultural diversity, bioethics, broad organismal biology, pharmacokinetics, environmental science, short-to-long temporal scales, diverse methodological approaches, and more, the field is necessarily interdisciplinary, as reflected in our special issue. At the time of writing there are four review articles and 19 research articles in the issue, and more will be added over time; a number of the authors of these articles will also speak at an upcoming eLife symposium on this topic. Collectively, these articles and the existing literature on evolutionary medicine (see, for example, Benton et al., 2021; Corbett et al., 2018; Gluckman et al., 2011; Moltzau Anderson and Horn, 2020; Stearns, 2012) illustrate the enormous breadth of this field and its potential to help advance our understandings of human health and the treatment of disease.

## Acknowledgements

It was my privilege to work with Dominique Soldati-Favre (Senior Editor), Ben Cooper, Vaughn Cooper, Sophie Helaine, Frank Kirchhoff, Paul Rainey, Antonis Rokas (Reviewing Editors), Amy Goldberg and Imroze Khan (Guest Editors) as an editorial team on this special issue. Detlef Weigel (Deputy Editor) was encouraging of the concept from the outset. The editorial team worked with numerous outstanding reviewers, whose great insights and collegiality benefitted both the consultative review process and the published articles. I also grateful to those colleagues who provided feedback on an earlier draft of this article. Finally, I would like to acknowledge Maria Guerreiro and other eLife editorial staff members for their expert contributions to this effort.
